# Multiparametric performance comparison of dental composites for clear aligner attachments

**DOI:** 10.1186/s12903-025-06623-w

**Published:** 2025-07-19

**Authors:** Yunlin Guan, Jiarong Xu, Junhong Qiu, Hao Cai, Wenxuan Xia, Zhou Ye, Ting Sang

**Affiliations:** 1 School of Stomatology, Jiangxi Medical College, Nanchang University, Nanchang, 330000 Jiangxi China; 2 Jiangxi Provincial Key Laboratory of Oral Diseases and Jiangxi Provincial Clinical Research Center for Oral Diseases, 688 Honggu North Avenue, Honggutan District, Nanchang, 330000 Jiangxi China; 3 Applied Oral Sciences and Community Dental Care, Faculty of Dentistry, The University of Hong Kong, Hong Kong S.A.R., China; 4 Department of Stomatology, Liuzhou Worker’s Hospital, Liuzhou, 545000 Guangxi Zhuang Autonomous Region China

**Keywords:** Clear aligner, Composite attachment, Color stability, Shear bond strength, Durability

## Abstract

**Background:**

As clear aligner technology (CAT) gains prominence, the performance of composite attachments - critical devices for optimizing aligner retention and tooth movement control - require systematic evaluation. This study assesses three light-cured composites (Filtek™ Z250 XT, Z350 XT, and P60; 3 M ESPE) regarding color stability, shear bond strength (SBS), and durability to establish evidence-based selection criteria.

**Methods:**

Attachments were bonded to mandibular premolars, simulating the clinical process, and materials were tested for color changes (after immersion in coffee, cola, or iced tea), SBS, and durability (wear volume, surface roughness, morphology, post-aging SBS). The data obtained from the study were statistically evaluated via the Shapiro-Wilk test, the Levene test, t-tests, one-way analysis of variance and chi-square test. A *p*-value < 0.05 was considered statistically significant.

**Results:**

Z250 showed significantly higher coffee - induced discoloration than Z350 (*p* < 0.05) and P60 (*p* < 0.01), exceeding clinical acceptability (ΔE_00_ ≥ 3.3). Z250 also emerged similar trends with cola and iced tea. Z350 exhibited the highest immediate SBS (*p* < 0.05 vs. Z250) that may cause enamel damage. P60 demonstrated superior wear resistance, with significantly lower surface roughness (Sq / Sa) than Z250 (*p* < 0.001) and Z350 (*p* < 0.01), and the smallest post-wear defect volume (*p* < 0.01 vs. Z250). The SBS differences in immediate groups were eliminated through aging treatment.

**Conclusions:**

Z250 underperformed in color stability, SBS, and durability versus Z350/P60, though demonstrated cost-effectiveness. Z350 offers outstanding color durability and higher SBS but risks enamel damage from interfacial delamination. P60 excels with color stability, acceptable adhesive remnants, and exceptional wear resistance, serving diverse clinical needs. Clinical decisions could prioritize P60 for function-aesthetic balance, with targeted Z350/Z250 use in special scenarios.

## Background

Over the past two decades, advancements in materials and technologies have significantly enhanced Clear Aligner Therapy (CAT). Thanks to the development of 3D imaging and computer-aided design/manufacturing (CAD/CAM) technology, notably, clear orthodontic appliances, such as Clear Aligners, first appeared in 1997. These appliances are uniquely designed, customized to fit each patient’s dental arch, and feature a high aesthetic appeal compared to traditional fixed orthodontic appliances [1]. Clear Aligners (CA) also possess advantages, including excellent hygiene and comfort, which are essential for patient satisfaction and long-term use [2]. And with the introduction of innovative materials, such as reversible memory materials and antibacterial coating technology, the efficiency and overall healthiness of CA has been further enhanced [3–5]. As a result, clear orthodontic appliances are increasingly gaining acceptance and preference among patients [6].

CA achieves precise tooth movement through resin attachments, which are specially designed, created by filling templates with light-cured composite resin materials, then securely bonded to the enamel surface using an adhesive system to form stable connections. The buckle structure is formed between the clear aligners and teeth, which improves the fixation of orthodontic appliance, increases the predictability and effectiveness of tooth movement [7]. Therefore, the application of resin attachments in CA is of paramount importance.

However, the market currently lacks a product design specifically tailored for CA attachments. Common materials such as resin and glass ionomer materials are primarily designed for dental hard tissue repair, their properties do not fully align with the unique requirements of attachment materials [8, 9]. The primary application requirements for clear aligner attachment materials include the following aspects: (a) Aesthetic Performance: Resin attachments, as protruding structures on the enamel surface, necessitates improved resistance to prevent discoloration over time. (b) Bonding Performance: Resin attachments must demonstrate reliable adhesion properties during orthodontic treatment to minimize detachment, thereby avoiding compromised treatment efficacy and increased clinical visits. Additionally, minimizing residual adhesive remnants during debonding is essential to prevent enamel damage [10]. (c) Durability: The effectiveness of clear aligner treatment depends on the structural integrity of the attachments, which are responsible for transmitting orthodontic forces and controlling tooth movement [11].

Correlatively, there is also a lack of standardized clinical guidelines for selecting materials. Orthodontists often rely on empirical clinical experience rather than objective data. Some recent studies in this field focus narrowly on isolated material properties rather than providing multiparametric evaluations [12–14], the evidentiary basis guiding orthodontists’ selection of attachment materials, consequently, demonstrates methodological constraints. Therefore, a multiplexed evaluation, accurately simulate clinically relevant conditions, is essential to identify suitable attachment materials.

Addressing this require, our work conducts a multiparametric performance comparison of Filtek™ Z250 XT, Z350 XT, P60 (3 M ESPE, St. Paul, MN, USA), three market-prevalent light-cured composite resin that widely used in clinical practice (Table 1). Selected for their biocompatibility, aesthetic versatility, ease of handling, and cost-effectiveness [15, 16], these materials were tested according to ISO 4049:2019, under clinically relevant conditions in vitro, to compare their color stability, shear bond strength (SBS), and durability to provide guidance for the selection of optimal attachment materials for CAT. The null hypothesis posits no significant differences among the three materials in these evaluated parameters.

**Table 1:** Information of materials applied in this study

Material	Manufacturer	Organic matrix	Fillers
Z250^a^	3 M ESPE	Bis-GMA, Bis-EMA, UDMA, TEGDMA^b^	0.01–3.5 μm zirconia/Silica (82 wt%)
Z350^a^	3 M ESPE	Bis-GMA, Bis-EMA, UDMA, TEGDMA^b^	5–20 nm silica nanofillers and 0.6–1.4 μm zirconia/Silica nanoclusters (78.5 wt%)
P60^a^	3 M ESPE	Bis-GMA, UDMA, Bis-EMA^b^	0.01–3.5 μm zirconia/Silica (83 wt%)

^a^ Z250 = Filtek™ Z250 XT; Z350 = Filtek™ Z350 XT; P60 = Filtek™ P60;

^b^ Bisphenol-A-glycidylmethacrylate, Bis-GMA; Bisphenol-A polyethylenglycol dietherdimethacrylate, Bis-EMA; Urethane dimethacrylate, UDMA; Trietyhlenglycol dimetacrylate, TEGDMA

## Methods

### Sample preparation

Total of 45 mandibular premolars (chosen for clinical availability from orthodontic extractions and standardized buccal anatomy minimizing substrate variability) were collected from Affiliated Stomatological Hospital of Nanchang University [13, 17].

The collected teeth were cleaned of debris, calculus, and soft tissues using dental scalers, followed by polishing with prophylaxis paste. Disinfection was performed by immersing the teeth in a solution of compound benzalkonium bromide disinfectant (LIRCON, pH = 5.5, benzalkonium bromide 27–33 g/L, Dezhou, Shandong, China) diluted in distilled water at a 1: 29 ratio for 72 h. After disinfection, the teeth were stored in purified water at 37 ℃ until use [18].

Each 10 premolars were embedded in gypsum to simulate dental arch morphology. The models were scanned using an iTero intraoral scanner (Align Technologies, San Jose, CA, USA), and 3Shape Ortho Analyzer software (3Shape A/S, Copenhagen, Denmark) was employed to design vertical rectangular attachments (4 × 2.5 × 2 mm) at the mid-coronal third of the buccal surfaces. STL digital models were exported and 3D-printed to create plastic dental models. Attachment templates were fabricated using a vacuum-forming machine (JG-206 Vacuum Former, Wuhan, Hubei, China) with 0.6 mm thermoplastic films.

The enamel surfaces were cleaned with alcohol swabs and dried with compressed air. The middle third of the buccal surfaces was etched with 35% phosphoric acid for 30 s, rinsed with water for 15 s, and air-dried. Adper™ Single Bond 2 adhesive (3 M ESPE, St. Paul, MN, USA) was applied and light-cured for 3 s using a dental curing unit (Woodpecker b-cure, Nanning, Guangxi, China).

**Fig. 1:**
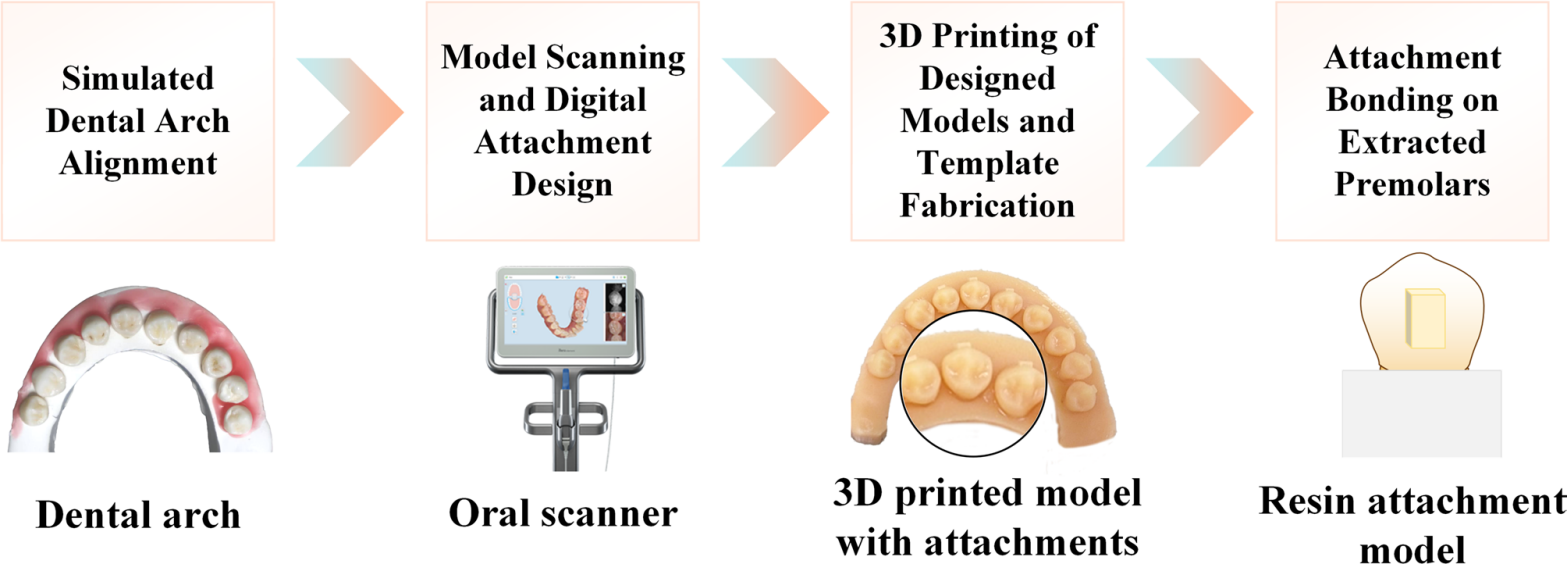
The fabrication process of tooth-attachment model

The attachment template was customized for each tooth as an individual unit. Based on the material of the filled attachment template, the groups were classified as follows: Group A: Filled with Filtek™ Z250 XT light-cured composite resin; Group B: Filled with Filtek™ Z350 XT light-cured composite resin; Group C: Filled with Filtek™ P60 light-cured composite resin. Individual templates were trimmed to fit single crowns. After resin filling, the templates were fully adapted to the tooth surfaces and light-cured (400 mW/cm² mode) for 40 s. After bonding, the models were stored in distilled water for 24 h to complete polymerization. These models were used for color stability and SBS testing (Fig. 1).

### Color stability evaluation

The resin attachment models (*n* = 5 for each material) of extracted teeth were separated from the dental arch and sequentially immersed in different colored solutions: (1) Coffee solution: Prepared by mixing 5 g of granulated coffee (Nestlé, Vevey, Vaud, Switzerland) with 50 mL of 100 ℃ ultrapure water, (2) Cola solution (Coca-Cola Company, Atlanta, Georgia, USA), (3) Iced tea solution (Wuhan Uni-President Foods Co., Ltd., Wuhan, Hubei, China). The samples along with 10 mL of each colored solution were placed in 20 mL glass vials and immersed in a water bath at 37 ℃. Each colored solution was used for an immersion period of 8 days, during which the samples were rinsed, and the solution was replaced daily [19]. Photographs of the resin attachment models of the extracted teeth were taken before immersion and after each solution immersion period (8 days, 16 days, 24 days) using a camera with fixed parameters: 5500 K color temperature, ISO 250, 20 cm distance and F36 white balance. All photographs were taken under standardized lighting with a blue background and in the same photography studio. The captured images were imported into Colormeter software (Konica Minolta, Inc., Tokyo, Japan) [13]. The surface of the attachments was divided into three equal regions: upper, middle, and lower. The intersection points of the diagonals in each region were selected to measure the L, a, and b values, and the average values were calculated. These values were input into the CIEDE2000 formula to calculate the color difference ΔE_00_, where ΔE_00_ ≥ 3.3 was considered clinically unacceptable color difference [20]. The CIEDE2000 formula is as follows:

$$\displaylines{\Delta {E_{00}} = \cr \sqrt {{{\left( {\frac{{\Delta L'}}{{{S_L}}}} \right)}^2} + {{\left( {\frac{{\Delta C'}}{{{S_C}}}} \right)}^2} + {{\left( {\frac{{\Delta h'}}{{{S_h}}}} \right)}^2} + R \cdot \left( {\frac{{\Delta C'}}{{{S_C}}}} \right) \cdot \left( {\frac{{\Delta h'}}{{{S_h}}}} \right)} \cr} $$

### Shear bond strength measurement

**Fig. 2:**
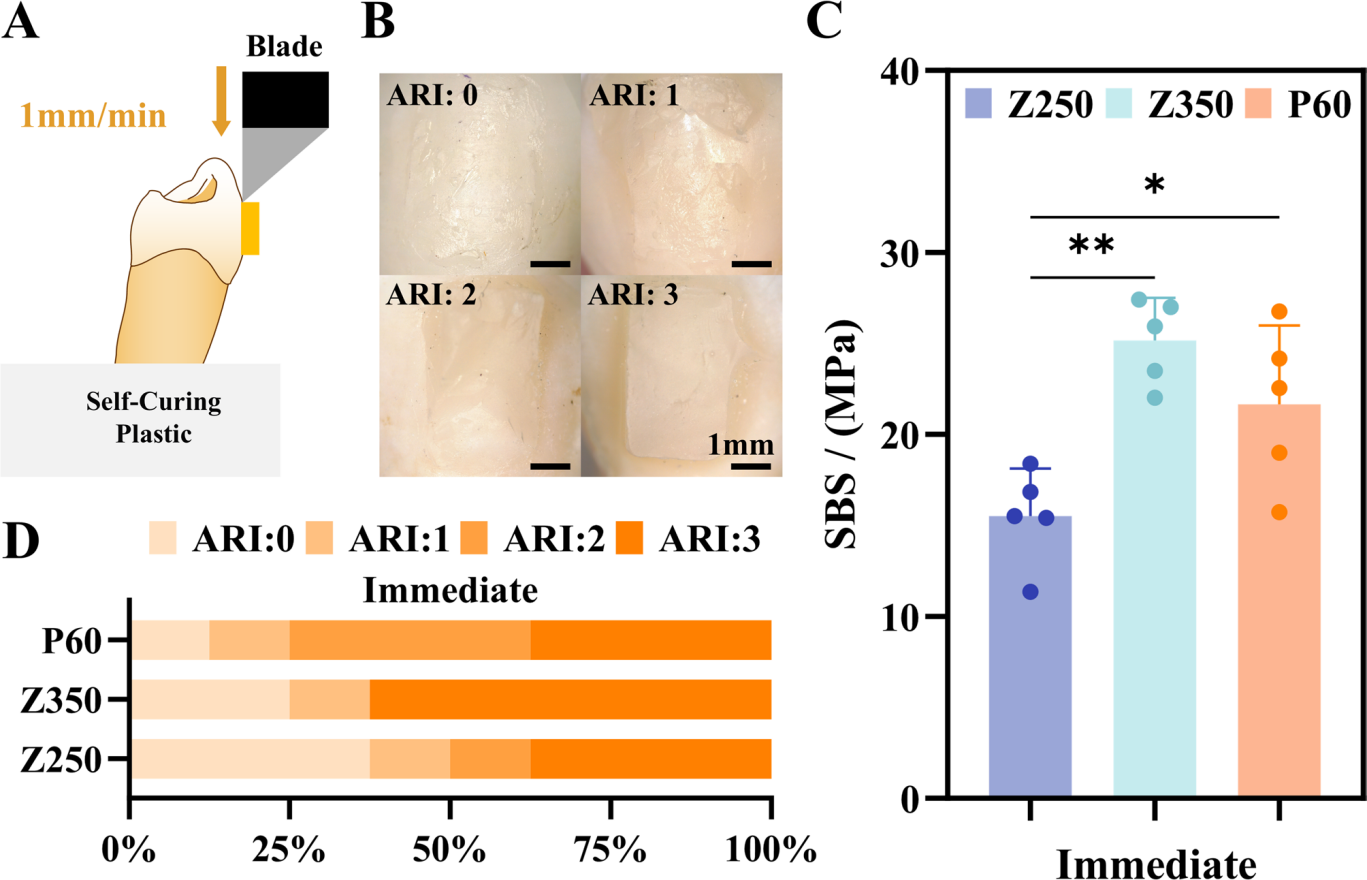
(**A**) The model for SBS test. (**B**) Representative images of classification of ARI: 0 represents the absence of residual resin on the enamel surface; 1 represents less than half of the residual resin remains; 2 represents more than half of the residual resin remains; 3 represents almost all residual resin remains. (**C**) Immediate SBS of three materials. (**D**) ARI results after SBS test

Each resin attachment model was embedded in self-curing plastic (*n* = 5 for each material), and the SBS was measured using an Instron universal testing machine (INSTRON 2343P8241, Norwood, Massachusetts, USA). The unloading force direction was parallel to the long axis of the attachment, and a blade-type loading structure was placed vertically between the rectangular resin attachment and the enamel surface. The loading speed was set at 1 mm/min, and the computer recorded the force value in Newtons (N) at the moment of attachment detachment (Fig. 2A) [21]. According to the attachment size data in the experimental design, the base area of the attachment was 10 mm², and the bond strength was calculated using the following formula: Bond Strength (MPa) = Maximum Load (N)/Attachment Base Area (mm²). After the SBS test, the enamel surface condition after attachment detachment and the resin bonding residual area on the enamel surface were observed under a stereomicroscope (Leica EZ4W, Wetzlar, Hesse, Germany) at 10 × magnification, and the adhesive remnant index (ARI) was recorded (Fig. 2B) [22].

### Durability experiment

#### Wear test

**Fig. 3:**
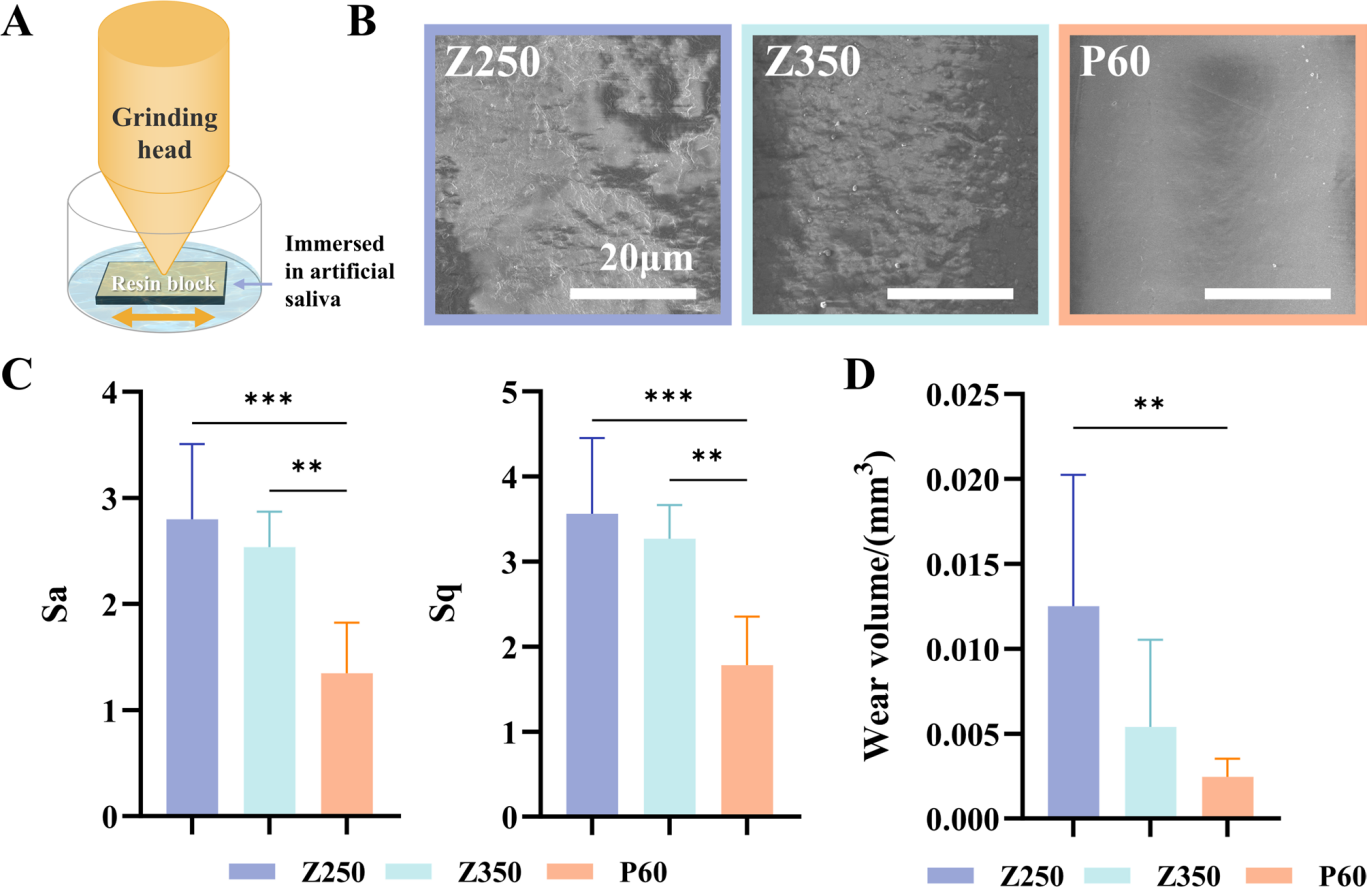
(**A**) An abrasion model simulating aligner insertion and removal in oral. (**B**) SEM pictures of materials’ surface after abrasion. (**C**) Post-wear surface roughness (Sq and Sa values). (**D**) Wear-induced defect volume

For the wear test of durability, silicone molds (7 × 4 × 1 mm) were filled with the three resin materials (*n* = 7 for each material), flattened using transparent films, and light-cured for 40 s. The cured resin blocks were adhesively bonded to plastic caps (diameter: 18 mm) for subsequent mechanical testing. 3 mL of artificial saliva were poured into a plastic cap fixed with a resin block, submerging the resin block, and placed on the stage of a simulated wear device. Under a 2.5 kg load, a metal stainless steel grinding head (diameter = 2 mm) was applied to the specimen, and horizontal relative motion wear was performed along a 3 mm linear path at a frequency of 1 Hz for a total of 1000 reciprocating cycles (Fig. 3A) [23], simulating the aligners’ insertion and removal process for one year. After the wear operation, the resin block was removed, rinsed for 1 min, and the surface water was blown dry. The surface of each specimen was recorded using an optical profiler (Bruker Corporation, Billerica, MA, USA). The surface roughness values Sa and Sq were directly obtained by detecting the surface of each specimen before and after wear using the optical profiler. The volume of the worn area was calculated based on the average pit depth after wear and the area of the shooting window, using the height level line of the unworn area as a reference. The surface morphology after wear was photographed under a stereomicroscope at 10 × magnification. The surface roughness and micro-morphology of the resin attachment after wear were observed using a scanning electron microscope (SEM) at 2000 × magnification (Acceleration voltage: 5.0 kV; Working distance: 20.0 μm) (Fig. 3B).

#### Aging test

**Fig. 4:**
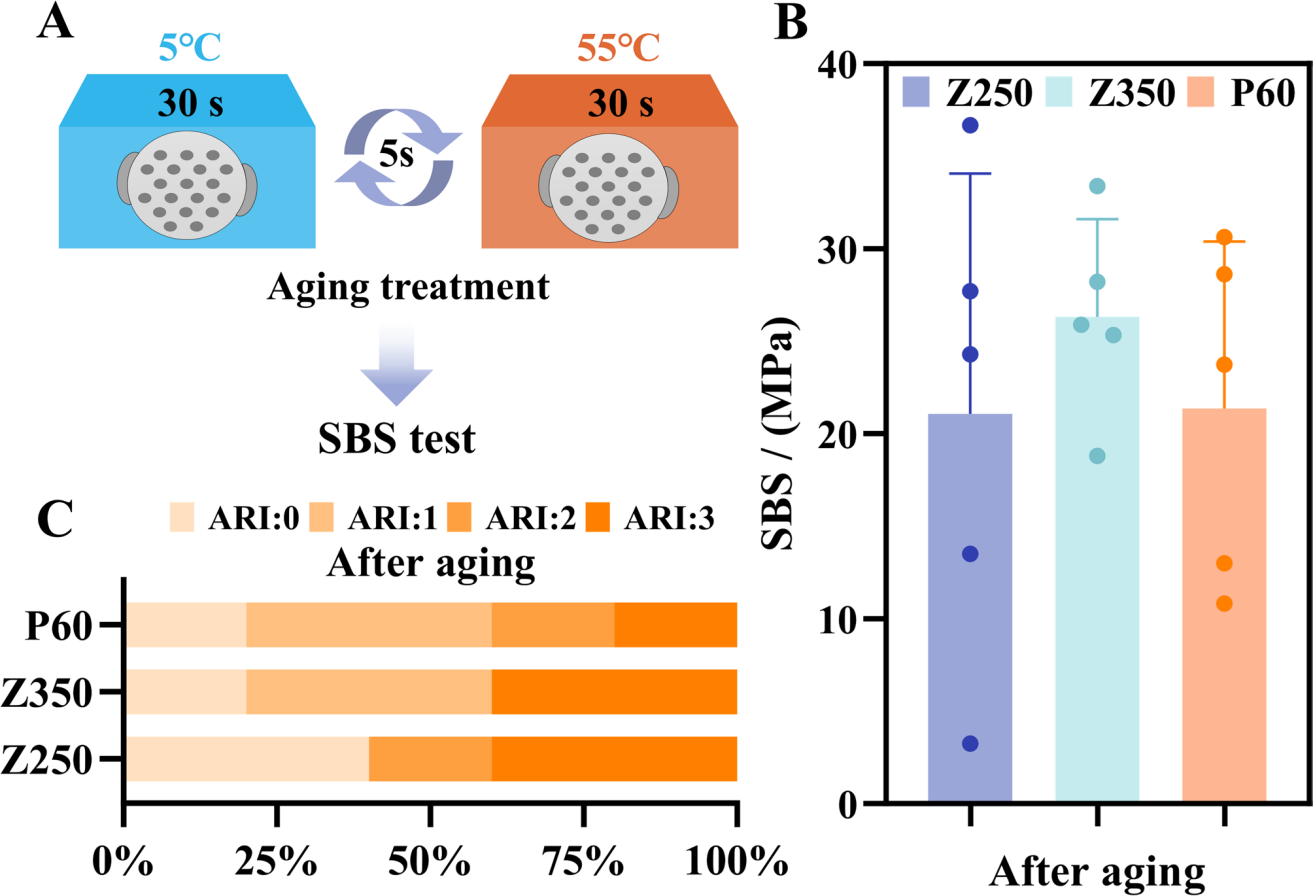
(**A**) Aging treatment conducted by thermal cycling. (**B**) SBS after aging. (**C**) Results of ARI

The resin attachment models (*n* = 5 for each material) were placed in a perforated metal container and aged in a thermo Neslab EX-7 recirculating bath (Marshall Scientific, Hampton, NH, USA), which involved 10,000 thermal cycles between 5 ℃ and 55 ℃ with a dwell time of 30 s and a transfer time of 5 s, simulating intraoral functional conditions for one year (Fig. 4A) [24]. After the aging treatment, the models were subsequently subjected to both the SBS experiment and ARI evaluation, following the standardized methodology described in previous sections.

### Statistical analysis

Using SPSS 25.0 software (IBM Corporation, Armonk, New York, USA), data were presented as “mean ± standard deviation”. Normality was assessed using the Shapiro-Wilk test, and homogeneity of variances was evaluated using Levene’s test. For comparisons between non-aged and aged groups of the same material, independent samples t-tests were conducted. One-way analysis of variance (ANOVA) was used for comparisons among different materials. A *p*-value < 0.05 was considered statistically significant. For non-normally distributed data (*p* < 0.05), Kruskal-Wallis H test was used for between-group comparisons. Categorical data, like ARI value, were analyzed using the chi-square test for independence (row × column chi-square test).

## Results

### Color stability evaluation

**Fig. 5:**
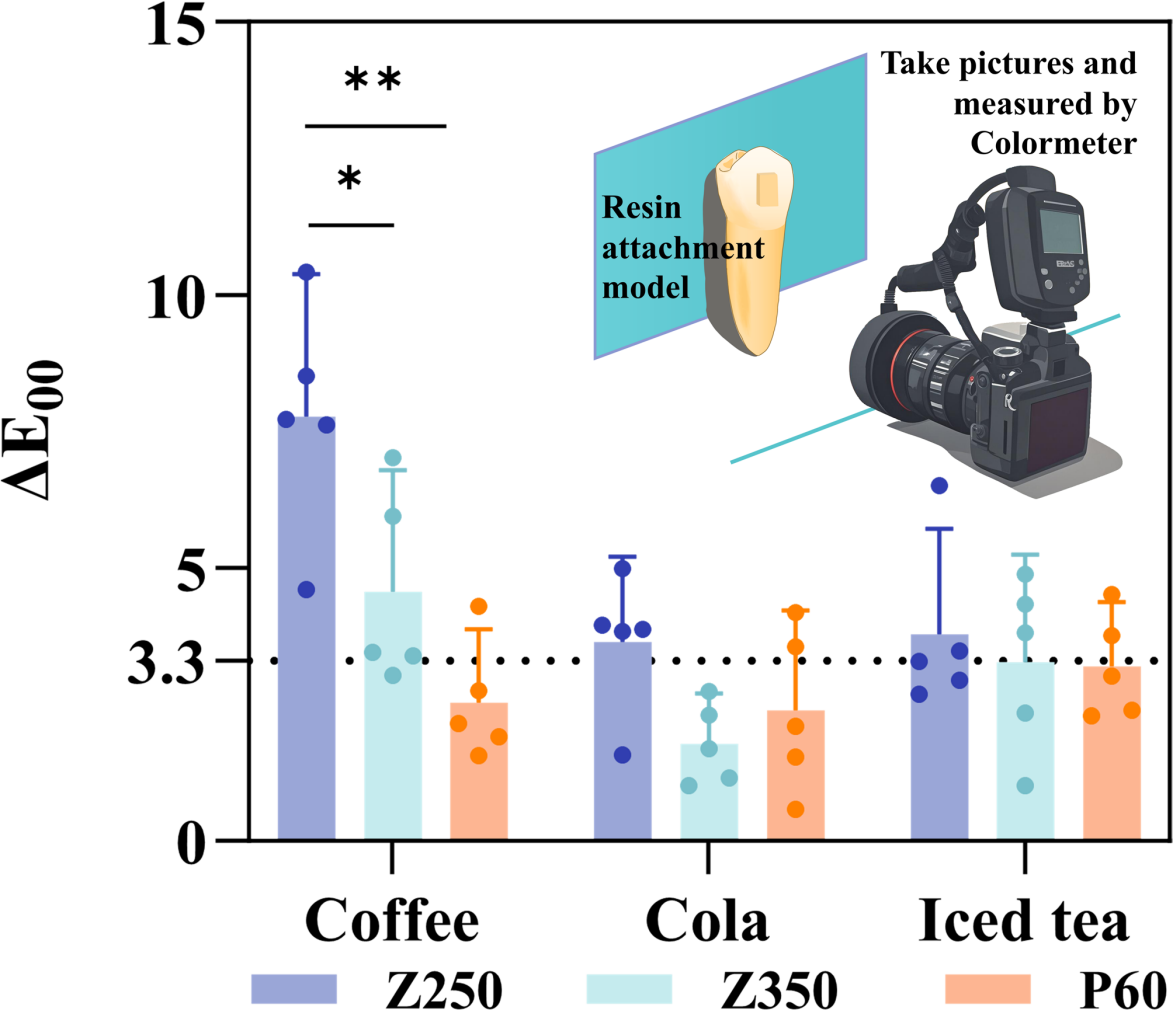
Color stability testing methodology and experimental outcomes

After 8 days of coffee immersion, the ΔE_00_ value of Z250 (7.787 ± 2.10) was significantly higher than that of Z350 (*p* = 0.030) and P60 (*p* = 0.001). The color changes of both Z250 and Z350 exceeded the clinically acceptable range (ΔE_00_ ≥ 3.3). After 8 days of immersion in cola (ΔE_00_ = 3.65 ± 1.25) and iced tea (ΔE_00_ = 3.795 ± 1.55), the color changes of Z250 also exceeded the clinically acceptable range, but there were no statistical significant differences among the groups (Table 2; Fig. 5).

**Table 2:** The ΔE_00_ of tested materials after immersion in coffee, cola, and iced tea

Materials	Coffee	Cola	Iced tea
**Z250**	7.78 ± 2.10^b, B^	3.65 ± 1.25^a, A^	3.78 ± 1.55^a, A^
**Z350**	4.57 ± 1.79^a, B^	1.79 ± 0.74^a, A^	3.28 ± 1.58^a, AB^
**P60**	2.54 ± 1.08^a, A^	2.39 ± 1.48^a, A^	3.20 ± 0.94^a, A^

^A, B^ Indicate statistically significant differences in the row

^a, b^ Indicate statistically significant differences in the column

Significant difference (*p* < 0.05)

### Shear bond strength measurement

The immediate measurement results showed that the SBS of Z350 was significantly higher than that of Z250 (*p* = 0.001). The SBS of P60 was also higher than that of Z250 (*p* = 0.027) (Fig. 2C). After the shear test, Z350 showed the most adhesive residue, but there were no statistically significant differences (*p* = 0.603) in the ARI results of the three materials in the immediate groups (Fig. 2D).

### Durability experiment

#### Wear test

The data from the optical profiler showed that the Sq and Sa values of P60 after wear were the smallest, with significant differences compared to Z250 and Z350, while the Sq and Sa values of Z250 were the largest (Fig. 3C). The SEM results showed that the surface of P60 after wear was the smoothest, while the surface of Z250 was the roughest, indicating that the actual observations were consistent with the measured values (Fig. 3B).

There was a statistically significant difference in the wear defect volume between Z250 and P60 (*p* = 0.007). Specifically, the wear defect volume of P60 was smaller than that of Z250. The wear defect volume of Z350 fell between the other two materials in terms of mean value (Fig. 3D).

#### Aging test

After the aging cycle, the order of SBS from high to low was consistent with the immediate measurement results, that is, Z350 had the highest SBS and Z250 had the lowest, but there were no significant differences among the groups. The SBS of each material after aging was higher than the immediate measurement results (Fig. 4B). P60 had the least adhesive residue in the after-aging group than others. Both Z350/P60’s adhesive residue decreased after aging, while that of Z250 was basically consistent with the non-aging group (Fig. 4C).

## Discussion

This study evaluated the color stability, SBS, and durability of three dental resin composites (Z250, Z350, P60) under simulated clinical conditions. Color stability testing involved immersing materials in coffee, cola, and iced tea especially at physiological temperatures. This study may have advantages in the construction of the experimental model. Compared to other studies using resin disks as samples, constructing a resin attachment model can better simulate the application scenario of clear aligner resin attachments in oral. The color difference of the resin material after staining with colored solution was higher in this study than in other studies [25]. The results of this evaluation revealed that Z250 exhibited a color difference exceeding the clinically acceptable range after soaking in all three colored solutions, with the largest color difference observed after 8 days of soaking in coffee. The phenomenon may be attributed to Z250’s micron-scale silica fillers (0.01–3.5 μm) and highest resin matrix content, which exhibits higher surface roughness and water absorption rate, enhancing adsorption capacity for water-soluble chromogens (e.g., coffee and tea) [26]. In contrast, Z350 incorporates densely packed nanoscale fillers that create a smoother surface and minimize resin matrix exposure. The superior color stability of Z350 observed in this study can be attributed to its structural advantage, which reduces pigment adsorption. P60 combines high-density fillers with reduction of resin matrix [27], that may minimize light scattering and shorten the propagation path within the composite. This reduction in scattering intensity might result in a less perceptible overall color change, enhancing the material’s aesthetic stability [28]. Other studies also exhibited a significant color difference in resin composite materials after colored solution immersion, especially in coffee [29]. Meanwhile, the composition of staining solutions has chance to influence outcomes. The acidity of cola may potentially weaken the filler-matrix interface in P60 composites due to hydrolysis of silane coupling agents, which could contribute to increased chromatic divergence compared to Z350. Conversely, the tannic acid and polyphenols in coffee or iced tea appear to interact preferentially with Z350 surfaces, potentially through hydrogen bonding with Si-OH groups, which may lead to comparatively more noticeable color discrepancies than those seen with P60 surfaces [30]. Therefore, for high esthetic requirement patients, Z250 should be used with caution due to its susceptibility to unacceptable color changes after exposure to common beverages. Z350 and P60 offer better color stability thanks to their filler structures. Clinicians should match dental resin materials with patients’ beverage consumption habits. For patients who frequently drink cola, it is advisable to choose alternatives to Z350 due to potential degradation risks in P60 and encourage them to reduce cola intake. Similarly, for those who regularly consume iced tea, refrain from using Z350, and suggest limiting intake of these beverages.

The shearing force experienced by resin attachments during debonding challenges typically differs from that experienced by caries restorations under occlusal forces. While attachments are generally not subjected to direct occlusal loading, their bonding interface is limited, often involving only a single surface of the cube bonded to enamel. In contrast, the resin block used in caries restoration is typically bonded on at least four surfaces to enamel or dentin. This results in a significant difference in bonding configuration between the two. Thus, when evaluating shear bond strength, our designed resin attachment model better simulates the clinical pattern for attachments. The immediate SBS results of all the three materials exceeded the clinically required range (6–8 MPa) [14], while Z250 showing the lowest strength, that may due to its lower filler ratio resulting in insufficient material rigidity and propensity to develop stress concentration points, predisposing cohesive failure. Simultaneously, the elevated matrix content within Z250 predisposes the material to intensified polymerization shrinkage stress, which mechanistically induces micrometer-scale interfacial cracks at the resin-enamel junction through anisotropic contraction patterns, thereby compromising SBS to some extent. Z350’s nanofiller arrangement displays enhanced rigidity than Z250, that achieve optimal SBS with compatible bonding agents [31]. P60’s hybrid fillers and highly crosslinked matrix tend to exhibit relatively superior inherent rigidity and comparatively minimal polymerization shrinkage, which may contribute to satisfactory mechanical hardness performance. Other studies, like Ribeiro et al., constructed a specific restoration model, reporting SBS for various resins within the range of 10–20 MPa [32]. Similarly to our experiment, Kircelli et al. designed rectangular attachments on the enamel surface, different from our study lies in its horizontally oriented rectangles, with the shearing surface corresponding to the long edge of the rectangle. Nevertheless, their reported SBS range for the resin attachments was consistent with our findings. However, a direct comparison is precluded due to the different resin materials used [14]. The ARI analysis revealed a predominant incidence of ARI score 0 in Z250 group, indicating a feasible monolithic debonding through bulk removal using a needle holder. It appears to reduce chairside time and minimize enamel damage. Conversely, Z350/P60 groups showed higher ARI scores suggesting difficulty in attachment removal, where controlled grinding protocols using high-speed turbines avoid catastrophic interfacial delamination-induced enamel fractures observed in direct peel-off methods.

In the durability testing, P60 exhibited the lowest roughness and wear volume, outperforming Z250 and Z350. These differences may be attributed to variations in filler characteristics, including size, shape, content, orientation, and distribution within the composite resin [33]. The observed superior wear resistance of P60 during wear cycles may be associated with its higher filler loading and potentially optimized filler-matrix coupling, demonstrating enhanced mechanical strength [34]. P60’s filler size and orientation, enabling uniform stress distribution and a “self-polishing” mechanism, forming enamel-like smooth surfaces. Its lower resin matrix content simultaneously appears to prevent localized excessive matrix abrasion. Conversely, Z250/Z350’s higher resin matrix content more likely lead to preferential matrix wear, exposing fillers and increasing roughness. Specifically, Z250’s micrometer-filler seems to create relatively heightened surface roughness post-wear compared to nanoscale-filled Z350. In clinical condition, P60’s relatively smooth surface after wear may reduce bacterial biofilm formation on the material surface to a certain extent [35], which is beneficial for maintaining oral hygiene and enamel health. Additionally, its small wear defect volume might indicate minimal wear deformation during aligner insertion and removal, which is advantageous for long-term force application and stability in CAT.

After the aging test, particularly treated with thermal cycling, all three materials demonstrated increased SBS, that may attribute to the post-curing effect of light-activated resins. It was likely Z250 had a lower initial resin matrix crosslinking degree, which increased significantly during aging, densifying its structure and boosting SBS. In comparison, Z350 and P60 had higher initial crosslinking, with less aging-induced change. Thus, Z250’s substantial SBS improvement bridged the gap, eliminating group differences. Hydroplasticization mediated by water-sorption phenomena facilitates a brittle-to-ductile transition in resin matrices through plasticizer-like molecular interactions, appears to enhance fracture resistance. Simultaneously, Ca²⁺ and PO_4_³⁻ ions in artificial saliva may deposit hydroxyapatite-like microlayers in ideal condition, filling micro cracks and reinforcing resin-enamel interfaces [36]. These findings suggest that restorations exhibit reduced risk of debonding during clinical aging.

Overall, P60 demonstrated an outstanding overall performance, while clinical observations indicate that it demonstrates limited adoption in routine practice, given its higher cost and limited color options. Z250 and Z350 remain predominant choices among prosthodontists and orthodontists based on empirical surveys. This may be because resins have traditionally been used for restoration, which emphasizes precise color matching. Therefore, Z350 and Z250 are more popular due to their broader shade selection. However, the application scenarios of orthodontic attachment differ from those of restorative resins. Aligner attachments are mostly used on posterior teeth, and even when used on anterior teeth, they are only applied temporarily or in small areas, resulting in less stringent requirements for shade aesthetics compared to restorative procedures. As such, P60’s overall performance is more suitable for orthodontic attachments. This study not only provides evidence-based support for challenging conventional material application norms but also provides clinical guidance for selecting attachment materials. It is suggested that clinicians prioritize P60 when seeking an optimal balance between functionality and aesthetics, employ Z350 to meet higher aesthetic requirements with more color options, and consider Z250 as an economical alternative.

Nevertheless, our work retains limitations characteristic of in vitro methodologies, including incomplete simulation of critical oral environmental factors such as dynamic pH fluctuations, bacterial biofilm interactions, and salivary enzyme activity [37]. To establish robust clinical correlations, future investigations should incorporate in *vivo* analyses coupled with controlled clinical trials, thereby validating material performance under physiologically relevant multi-factor oral environments.

## Conclusions

This study conclusively rejected the null hypothesis, demonstrating statistically significant variations in color stability, SBS, and durability across the tested materials. Z250 underperformed than Z350 and P60 in terms of color stability, shear bond strength, and durability tests. Z350 exhibits superior aesthetic properties and achieves effective bonding through higher SBS, however, the predominant presence of adhesive remnant at the failure interface indicates that direct interfacial delamination poses a higher risk of enamel damage, whereas meticulous removal using a high-speed turbine handpiece can mitigate such iatrogenic risks. P60 excels with clinically acceptable color stability, residual adhesive remnants and exceptional wear resistance, suggesting superior suitability for common orthodontic attachments requiring long-term functional durability and aesthetic preservation. Clinical decision-making should be guided by a three-dimensional framework encompassing aesthetic demand intensity, treatment duration, and cost sensitivity, prioritizing P60 to achieve an optimal balance between function and aesthetic. Targeted application of Z350/Z250 is recommended for specific clinical scenarios, complemented by standardized bonding protocols and patient behavior management to optimize treatment outcomes.

## Data Availability

The data supporting the findings of this study are available from the corresponding author upon reasonable request.
